# Identifying CDC7 as a synergistic target of chemotherapy in resistant small-cell lung cancer via CRISPR/Cas9 screening

**DOI:** 10.1038/s41420-023-01315-2

**Published:** 2023-02-02

**Authors:** Ling Deng, Li Yang, Shuhan Zhu, Man Li, Yu Wang, Xiaolong Cao, Qiongyao Wang, Linlang Guo

**Affiliations:** 1 Department of Pathology, Zhujiang Hospital, Southern Medical University, Guangzhou, China; 2 Clinical Oncology Center, The University of Hong Kong - Shenzhen Hospital, Shenzhen, China; 3 Division of Laboratory Medicine, Zhujiang Hospital, Southern Medical University, Guangzhou, China; 4 Translational Medicine Research Center, Zhujiang Hospital, Southern Medical University, Guangzhou, China; 5 Department of Oncology, Zhujiang Hospital, Southern Medical University, Guangzhou, China

**Keywords:** Small-cell lung cancer, Target identification

## Abstract

There is currently a lack of efficacious treatments for patients with chemo-resistant small-cell lung cancer (SCLC), leading to poor prognoses. We examined a chemo-resistant SCLC cell line using genome-wide CRISPR/Cas9 screening and identified serine/threonine kinase cell division cycle 7 (*CDC7*) as a potential synergistic target. Silencing *CDC7* in chemo-resistant SCLC cells decreased the IC_50_ and improved the efficacy of chemotherapy. Based on the highest single agent model, the CDC7 inhibitor XL413 had a synergistic effect with both cisplatin and etoposide in chemo-resistant SCLC cells, but had no such effect in chemo-sensitive SCLC cells; the combination of XL413 and chemotherapy significantly inhibited cell growth. Western blot and flow cytometry showed that the combined treatments increased apoptosis, whereas XL413 alone had little effect on apoptosis. An analysis of cell cycle and cyclin protein levels indicated that the combination of XL413 and chemotherapy-induced G1/S phase arrest and DNA damage in chemo-resistant SCLC cells. Xenografted tumor and histoculture drug response assays using patient-derived xenografts showed that XL413 improved the efficacy of chemotherapy in vivo and with SCLC tissues. These results suggest that XL413 exerts a synergistic effect with chemotherapy on chemo-resistant SCLC.

## Introduction

Small-cell lung cancer (SCLC) accounts for 15% of all lung cancers and is one of the most malignant tumors worldwide due to its poor prognosis [1]. Currently, first-line therapies for SCLC include cisplatin (DDP) and etoposide (VP16). The majority of patients with SCLC initially respond well to chemotherapy; however, chemo-resistant tumors tend to emerge shortly after therapy, resulting in relapse and poor survival [2]. Immune checkpoint inhibitors have been proposed as an option for the treatment of SCLC; however, the response rate to these is limited to approximately 15% of patients [3, 4]. Therefore, identifying novel therapies for patients with chemo-resistance is crucial to improve the prognoses of SCLC.

One solution to chemo-resistance is the combination of chemotherapy with target therapy. There are various approaches to identify possible targets, and CRISPR/Cas9-based gene knockout screening is a productive method for the identification of genes that act differently under various conditions [5, 6]. Based on the sgRNA carried, cells transfected with the CRISPR/Cas9 sgRNA library either increase or decrease in number as a result of treatment. Population changes between treatment and control groups can be used to identify negatively enriched sgRNAs to serve as potential synergistic targets because cells carrying such sgRNAs are less likely to survive treatment [7]. Such CRISPR/Cas9 library screening has identified FGF21 as a combinatorial therapeutic strategy for sorafenib-resistant hepatocellular carcinoma [8]. It also helped to identify the therapeutic significance of a FACT inhibitor in hedgehog-driven cancers [9]. Therefore, a CRISPR/Cas9 library is a practical tool for target therapy identification.

To explore potential targets for use in synergistic treatment with chemotherapy, we performed genome-wide CRISPR/Cas9 knockout screening of resistant SCLC cells with and without DDP and VP16 treatments. The results identified that serine/threonine kinase cell division cycle 7 (*CDC7*; also known as *DBF4*-dependent kinase) is a potential synergistic target. CDC7 is activated by binding to DBF4B, and it phosphorylates minichromosome maintenance-2 (MCM2) to initiate DNA synthesis [10]. CDC7 inhibitors have shown antitumor effects in various cancers [11, 12]; however, no study has explored the role of CDC7 and its inhibition in SCLC. The synergistic effect of CDC7 inhibitors and chemotherapy could help overcome chemo-resistance in SCLC treatment and improve the prognoses of patients.

## Results

### CRISPR/Cas9 library screening identifies CDC7 as a potential synergistic target in chemo-resistant SCLC

**Fig. 1:**
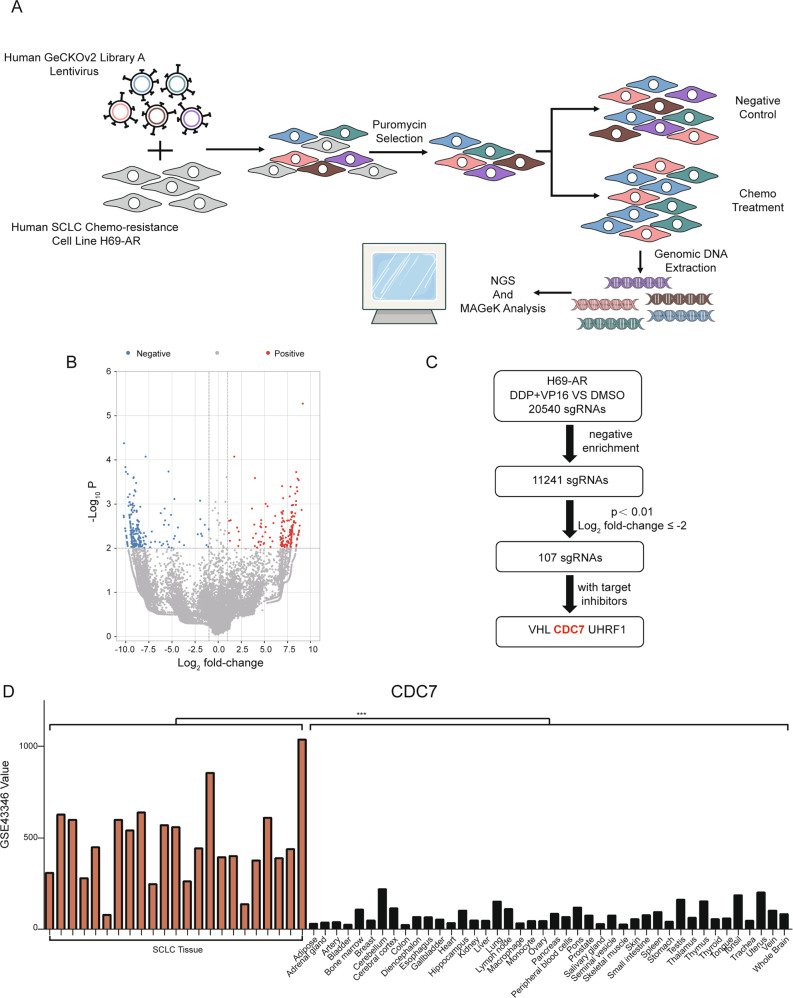
CRISPR/Cas9 screening identifies CDC7 as a potential synergistic target for chemo-resistant SCLC. **A** Schematic of the screening process using resistant SCLC cells H69-AR with GeCKOv2 library. **B** Volcano plot of differentially enriched sgRNAs between negative treatment and chemo-treatment. **C** Workflow of potential synergistic targets identification. **D** Expression of *CDC7* in SCLC and normal tissues from GSE43346. Independent sample t-test were used to examine statistical significance. *p < 0.05, **p < 0.01, ***p < 0.001.

To identify potential gene targets that synergize with chemotherapy in SCLC treatment, we analyzed data acquired from the in vitro CRISPR/Cas9 screening of the chemo-resistant SCLC cell line H69-AR (Figs. 1A and S1A). In total, 20 540 sgRNAs were detected in DDP and VP16-treated vs. DMSO-treated cells. A total of 11 241 sgRNAs was negative enriched and thus could be potential synergistic targets with chemotherapy (Table S1). A total of 107 sgRNAs had a log_2_ fold-change ≤ −2 and *p* < 0.01; three sgRNAs corresponded to genes with currently available targeted inhibitors, including *CDC7*, *VHL*, and *UHRF1* (Fig. 1B, C). Using data from GSE43346, we analyzed the expression levels of these three genes in SCLC and normal tissues. We found that *CDC7* and *UHRF1* were expressed at significantly higher expression levels in SCLC tissues than in normal tissues (*p* < 0.001 and *p* < 0.05, respectively). Meanwhile, the expression level of *VHL* showed no significant difference between the two groups (Figs. 1D and S1C, D). CDC7 inhibitors have been successfully administered to patients with solid tumors [13, 14], whereas UHRF1 inhibitors require further studies to determine their application in cancer treatment. These data indicated that CDC7 is a potential synergistic target with chemotherapy in chemo-resistant SCLC.

### Silencing *CDC7* improves chemotherapeutic efficacy in chemo-resistant SCLC

**Fig. 2:**
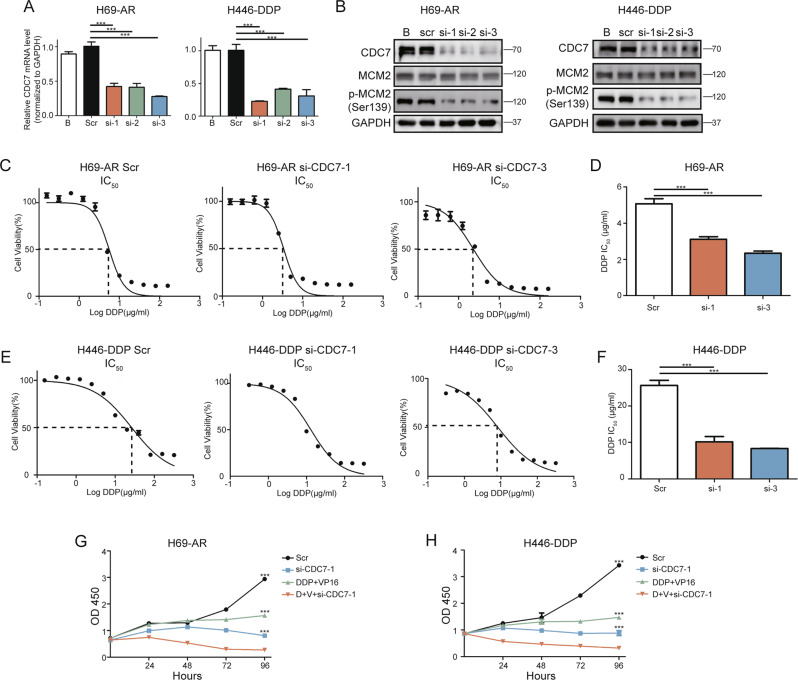
*CDC7* silencing improves chemo-treatment efficiency in resistant SCLC cells. **A** qRT-PCR analyses of *CDC7* expression in H69-AR(left) and H446-DDP(right) 48 h after transfected with *CDC7* siRNA. **B** Western blot analyses of CDC7 and MCM2 in H69-AR(left) and H446-DDP(right) 48 h after transfected with *CDC7* siRNA. IC_50_ and the statistics of IC_50_ of DDP in H69-AR (**C**, **D**) and H446-DDP (**E**, **F**) transfected with scramble or si-*CDC7*. Cell viability of H69-AR (**G**) and H446-DDP (**H**) transfected with si-*CDC7* using CCK-8 assay. All data are representative of three independent experiments (mean ± SEM).

To further evaluate the role of CDC7 in chemo-resistant SCLC, we silenced *CDC7* using small interfering RNA (siRNA) in H69-AR and H446-DDP cells. The chemo-resistant SCLC cell line H446-DDP was established from NCI-H446 cells that we had previously studied [15, 16] in our laboratory (Fig. S1A). After siRNA transfection into H69-AR and H446-DPP cells, qRT-PCR was conducted to confirm that *CDC7* mRNA (Fig. 2A) and protein levels were downregulated in both H69-AR and H446-DDP cells. Likewise, MCM2 phosphorylation showed that the activity of CDC7 was downregulated (Fig. 2B).

To determine the role of CDC7 in the chemotherapeutic response, we compared the IC_50_ values of DDP and VP16 with and without *CDC7* siRNAs in chemo-resistant SCLC cell lines. *CDC7* silencing significantly reduced the IC_50_ values of DDP and VP16 in both H69-AR and H446-DDP cells (Figs. 2C–F, S2A–D). Cell growth evaluations using Cell Counting Kit-8 showed that chemotherapy and *CDC7* silencing individually inhibited cell growth; however, the combination of these treatments was associated with a higher inhibition rate than either treatment alone (Figs. 2G, H, S2E, F). These data indicate that silencing *CDC7* improves chemotherapeutic efficacy in chemo-resistant SCLC cells.

### CDC7 inhibitor XL413 shows a synergistic effect with chemotherapy in vitro

**Fig. 3:**
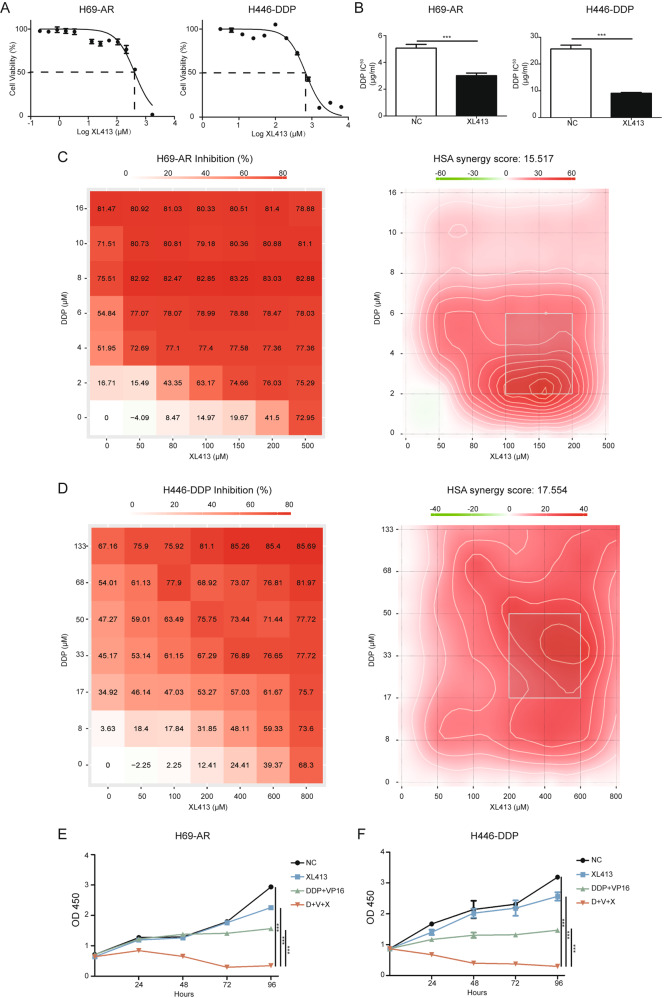
CDC7 inhibitor XL413 shows synergistic effect with chemo-treatment in resistant SCLC cells. **A** IC_50_ of XL413 in H69-AR(left) and H446-DDP(right) are 416.8 μM and 681.3 μM. **B** Comparison of DDP IC_50_ with or without XL413 in H69-AR(left, XL413 50 μM) and H446-DDP(right, 80 μM). **C** Cell viability matrices of DDP and XL413 in H69-AR(left) and the HSA model heatmap(right). **D** Cell viability matrices of DDP and XL413 in H446-DDP(left) and the HSA model heatmap(right). **E** Cell viability of H69-AR treated with negative control, XL413(50 μM), DDP, and VP16(1 μg/ml, 100 μg/ml) and combined treatment using CCK-8 assay. **F** Cell viability of H446-DDP treated with negative control, XL413(80 μM), DDP, and VP16(5 μg/ml, 80 μg/ml) and combined treatment using CCK assay.

To assess the potential of CDC7 as a clinical therapeutic target, we treated H69-AR and H446-DDP cells with the CDC7 inhibitor XL413. XL413 had a high IC_50_ value in both H69-AR and H446-DDP cells (416.8 μM and 681.3 μM, respectively, Fig. 3A), but a low dose of XL413 significantly reduced the IC_50_ values of DDP and VP16 in resistant SCLC cell lines (Figs. 3B, S3A, B). Next, we measured the potential synergistic effects between XL413 and chemotherapy in chemo-resistant SCLC cell lines. Using SynergyFinder version 2.0, we calculated and assessed cell viability using matrices with increasing concentrations of XL413, DDP, or VP16. Using the highest single agent (HSA) model, we evaluated the synergistic effects of XL413 and chemotherapy as follows: a score > 10 suggested a potentially synergistic effect between the two drugs [17, 18]. We found that the synergy scores of XL413/DDP and XL413/VP16 were >10 in both H69-AR and H446-DDP cells (Figs. 3C, D, S3C–F). Cell growth analysis showed that the combination of XL413 and chemotherapy had a greater and significant inhibitory effect compared to that of the negative control, XL413, and chemotherapy groups (Fig. 3E, F). Overall, the CDC7 inhibitor XL413 showed a synergistic effect with chemotherapy in chemo-resistant SCLC cell lines.

We further assessed whether XL413 would show a synergistic effect with chemotherapy in chemo-sensitive SCLC cell lines. The IC_50_ values of XL413 in the chemo-sensitive cell lines NCI-H69 and NCI-H446 were 485.1 μM and 365.1 μM, respectively (Fig. S4A). In contrast to the results using chemo-resistant cells, a low dose of XL413 failed to significantly reduce the IC_50_ of DDP and VP16 in NCI-H69 and NCI-H446 cells (Fig. S4B). We also evaluated the synergistic effect between XL413 and chemotherapy in chemo-sensitive SCLC cell lines using the HSA model. The synergy score suggested that XL413 had no synergistic effect when XL413 and chemotherapy were used NCI-H69 cells (Fig. S4C, D). However, in NCI-H446 cells, the synergy score indicated that XL413 had no synergistic effect with DDP but that it did have a potentially synergistic effect with VP16 (Fig. S4E, F). These data indicated that XL413 had no significant synergistic effect with chemotherapy in chemo-sensitive SCLC cell lines.

### XL413 promotes chemotherapy-induced apoptosis in chemo-resistant SCLC

**Fig. 4:**
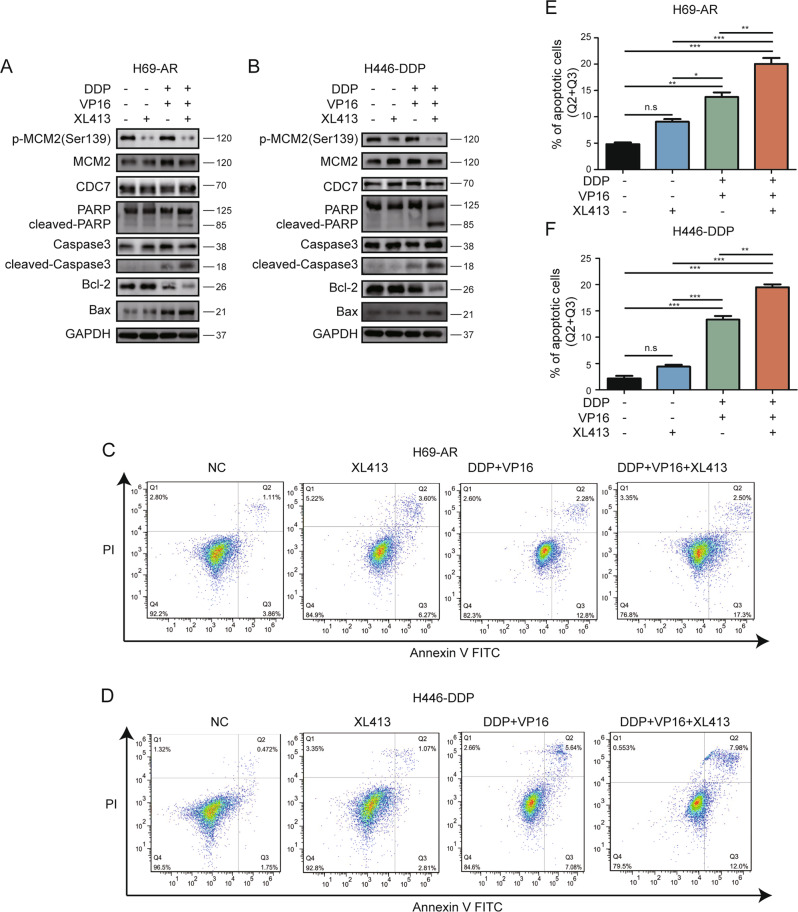
XL413 increases apoptosis induced by chemo-treatment in resistant SCLC cells. **A** Western blot analyses of apoptotic related proteins in H69-AR treated with negative control, XL413(50 μM), DDP and VP16(1 μg/ml, 100 μg/ml) and combined treatment for 48 h. **B** Western blot analyses of apoptotic related proteins in H446-DDP treated with negative control, XL413(80 μM), DDP, and VP16(5 μg/ml, 80 μg/ml) and combined treatment for 48 h. **C** Apoptotic flow cytometry of H69-AR with indicated treatment for 48 h. **D** Apoptotic flow cytometry of H446-DDP with indicated treatment for 48 h. Apoptotic cells statistical analyses of H69-AR (**E**) and H446-DDP (**F**).

We analyzed the levels of apoptotic proteins in cells treated with XL413, chemotherapy, or XL413 combined with chemotherapy. A low dose of XL413 alone did not increase the levels of apoptotic proteins, including cleaved poly (ADP-ribose) polymerase (PARP), cleaved caspase-3, and Bcl-2-associated X (Bax). In addition, the level of the anti-apoptotic protein Bcl-2 was not significantly decreased. Chemotherapy somewhat upregulated apoptotic protein levels and downregulated Bcl-2 levels, whereas the combined treatment significantly upregulated apoptotic protein levels and downregulated Bcl-2 levels in both H69-AR and H446-DDP cells (Fig. 4A, B).

We further assessed the proportion of apoptotic cells using flow cytometry. The apoptotic proportion of H69-AR cells did not significantly differ between the negative control and XL413 groups. Consistent with the results from the apoptotic protein analysis, the combined treatment group had the highest proportion of apoptotic cells of the four groups (Fig. 4C, E). Similar results were observed with H446-DDP cells (Fig. 4D, F).

To further confirm the role of CDC7 in chemotherapy-induced apoptosis, we performed flow cytometry on chemo-resistant SCLC cell lines transfected with *CDC7* siRNA. The results showed that silencing of *CDC7* combined with chemotherapy significantly induced apoptosis in chemo-resistant SCLC cell lines (Fig. S5A–D). These data suggest that silencing and inhibiting CDC7 promote chemotherapy-induced SCLC cell apoptosis in vitro.

### XL413 aggravates cell cycle arrest in chemo-treated resistant SCLC cell lines

**Fig. 5:**
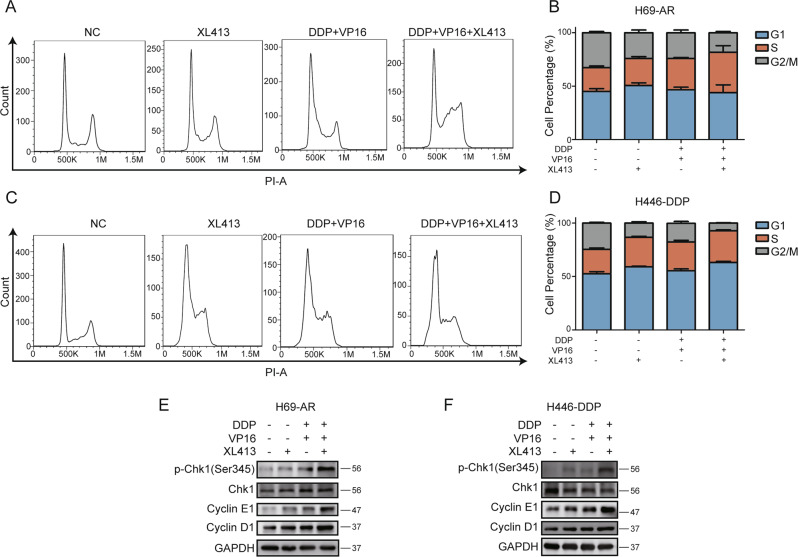
XL413 and chemo-treatment induce G1/S arrest in resistant SCLC cells. **A** Cell cycle flow cytometric analyses of H69-AR treated with negative control, XL413(50 μM), DDP, and VP16 (1 μg/ml, 100 μg/ml) and combined treatment for 48 h. **B** Quantification of cell percentage for each cell cycle in (A) and two more independent experiments. **C** Cell cycle flow cytometric analysis of H446-DDP treated with negative control, XL413 (80 μM), DDP, and VP16 (5 μg/ml, 80 μg/ml) and combined treatment for 48 h. **D** Quantification of cell percentage for each cell cycle in **B** and two more independent experiments. Western blot analysis of phosphorylated Chk1, cyclin E1, cyclin D1, and GAPDH in H69-AR (**E**) and H446-DDP (**F**) treated with XL413 and chemo-treatment alone or combined treatment for 48 h.

Since CDC7 is one of the key regulators of the cell cycle, we further analyzed cell cycle progression after XL413 and chemotherapy treatments. Cell cycle flow cytometry showed that XL413 caused a decrease in the G2/M population and an increase in the G1 and S phase populations of H69-AR cells. Chemotherapy alone mainly caused an accumulation of the S phase population and a reduction in the G2/M phase population. Similarly, XL413 combined with chemotherapy caused an accumulation of the S phase population and a reduction in the G2/M population (Fig. 5A, B). The individually treated XL413 and chemotherapy groups showed similar cell cycle arrest profiles in H446-DDP cells. Meanwhile, the combined therapy group showed the accumulation of the G1 and S phase populations (Fig. 5C, D).

We further analyzed cyclin protein levels and found that expression levels of cyclin D1 and cyclin E1 were upregulated in the treatment groups. Compared with those in individually treated XL413 and chemotherapy groups, the combined treatment group had higher cyclin protein levels in chemo-resistant SCLC cell lines. Thus, silencing *CDC7* had an effect on the cell cycle that was similar to that of inhibiting CDC7 (Fig. S6A–D).

We also analyzed the phosphorylation status of checkpoint kinase 1 (Chk1) to determine the level of DNA damage induced by cell cycle arrest. The results showed the upregulation of phosphorylated Chk1 levels in the treatment groups, particularly in the combined treatment group (Fig. 5E, F). These data indicated that CDC7 inhibition aggravated G1/S phase arrest and DNA damage in chemo-treated resistant SCLC cells.

### CDC7 inhibitor XL413 improves chemotherapeutic efficacy in vivo

**Fig. 6:**
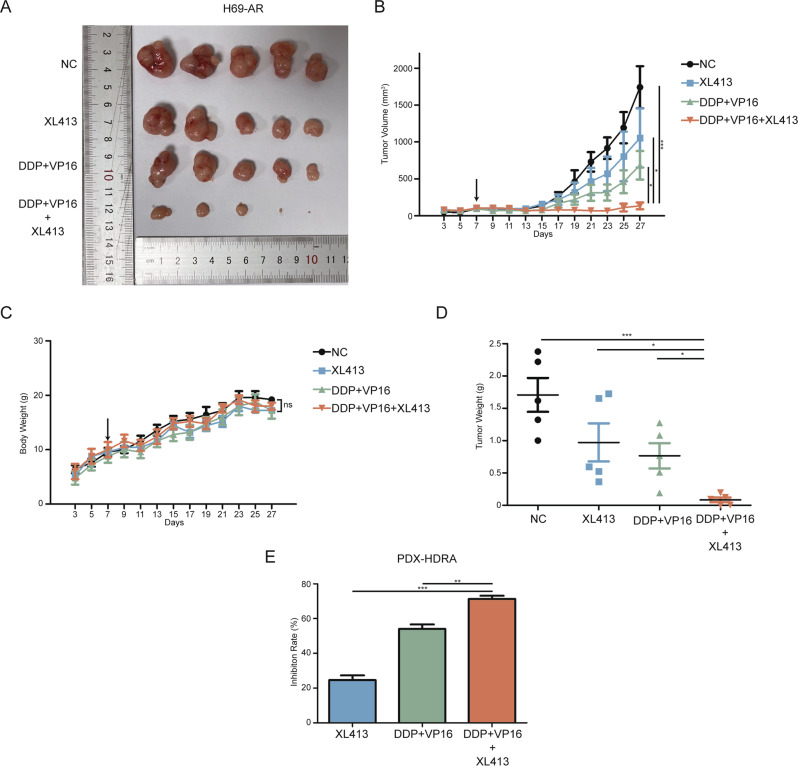
Combined treatment of XL413 and chemo suppresses resistant SCLC tumor growth in vivo. **A** Images of H69-AR xenograft tumors after XL413 (20 mg/kg) and chemo-therapy (2.5 mg/kg DDP and 4 mg/kg VP16) alone or combined treatment. **B** Tumor volume curve of xenograft tumors. The arrow indicated the start of treatments. **C** Body weight curve of H69-AR xenograft mice. The arrow indicated the start of treatments. **D** Tumor weight scatter plot of H69-AR xenograft model. **E** Inhibition rate of XL413 and chemo-therapy alone or combined treatment calculated via PDX HDRA.

To assess the potential of XL413 to be used as a clinical therapeutic target, we subcutaneously injected H69-AR cells into nude mice. Once the average tumor volume reached 100 mm^3^, we randomly divided the mice into four treatment groups (Fig. 6A). The combined treatment group showed significantly inhibited tumor growth, whereas the individual chemotherapy and XL413 treatments resulted in moderately inhibited tumor growth (Fig. 6B). Mouse body weight data showed that none of the treatments had significant side effects regarding weight (Fig. 6C). The weights of the resected tumors indicated significant tumor growth suppression in the combined treatment group (Fig. 6D).

To evaluate the efficacy of XL413 combined with chemotherapy, we performed a histoculture drug response assay (HDRA) on patient-derived xenograft (PDX) SCLC tumors. We found that XL413 treatment alone had the lowest inhibition rate and that combined treatment had the highest inhibition rate (Fig. 6E). These data indicated that XL413 improves chemotherapeutic efficacy in chemo-resistant SCLC xenograft models and thus has promising applications in SCLC therapy.

## Discussion

Therapeutic strategies for SCLC have shown no significant improvements in recent decades, especially for chemo-resistant SCLC. The search for effective molecular targets for second-line SCLC therapy has thus been an urgent topic in research. CRISPR/Cas9 is a productive method for high-throughput screening under various selective conditions and has been used to identify molecular subtypes and new therapeutic targets in SCLC; however, the sgRNA libraries used in these studies were not genome-wide. Researchers identified POU2F3 as a master regulator of SCLC by using a CRISPR/Cas9 library targeting 1427 DNA-binding domains of human transcription factors [19]. The druggable genome sgRNA library that these researchers used contained sgRNAs targeting approximately 750–5000 genes encoding targets [20, 21]. Our study was the first to use a genome-wide CRISPR/Cas9 screening system, which contained 123,411 sgRNAs targeting 19,050 genes, to identify targets for synergistic effects with chemotherapy in chemo-resistant SCLC. Based on our screening data and the fact that cell cycle inhibitors were hits for SCLC therapy, we focused on the role of CDC7 in chemo-resistant SCLC. We demonstrated that both silencing and inhibiting CDC7 improved the efficacy of chemotherapy in vitro, leading to cell apoptosis and cell cycle arrest. The results of xenograft tumor models and HDRAs using PDXs indicated that combined treatment with a CDC7 inhibitor and chemotherapy is effective in mouse models and patient-derived tissues. These results point to a promising clinical application of this treatment modality.

Preclinical models are effective tools for assessing the efficacy of novel antitumor strategies before clinical trials. In particular, PDXs have been shown to be useful in evaluating the tolerability and efficacy of new agents and treatment strategies prior to patient trials [22, 23]. However, acquiring SCLC tissues is difficult owing to the short surgery window for SCLC, and efficacy assessments using a PDX model are time-consuming. One productive method for utilizing PDX tissues and obtaining preliminary treatment efficacy data is HDRAs, which have been used to assess treatment efficacy for various solid tumors, including those of pancreatic cancer, cholangiocarcinoma, and non-small-cell lung cancer [24–26], but these have not been used for SCLC. Given the potential of our findings, which made use of data from a xenograft model and HDRA using a PDX, we anticipate the emergence of preclinical models and clinical applications targeting CDC7 in future studies.

Cell cycle inhibitors have shown antitumor efficacy for many tumors. The CDK4/6 inhibitor trilaciclib was added to first-line therapy for SCLC as a protector of chemotherapy-induced myelosuppression [27, 28]. Trilaciclib enhances the antitumor efficacy of the combination of chemotherapy and immune checkpoint inhibitors; however, no improvements in antitumor activity were shown with trilaciclib and chemotherapy. Trilaciclib acts on HSPCs and T cells instead of tumor cells in SCLC, but according to our results, XL413 was able to induce cell cycle arrest and DNA damage in SCLC cells. One possible explanation for this difference is that CDK4/6 modulates the cell cycle via Rb, encoded by a gene that is commonly mutated in SCLC, in the G1 phase, and CDC7 directly induces DNA synthesis in the S phase.

Given the key role of CDC7 in the cell cycle and DNA synthesis [29], many inhibitors are being developed to serve as antitumor agents. Targeting CDC7 induces DNA replication stress and shows therapeutic effects when applied either alone [11, 12] or in combination with other treatments [29, 30]. Our results showed that a CDC7 inhibitor combined with chemotherapy can induce cell cycle arrest and apoptosis, but more evidence is needed to elucidate the mechanisms linking these two treatments. The CDC7 inhibitor XL413 used in this study can inhibit CDC7 activity, as demonstrated based on phosphorylated MCM2 levels. It is known that DBF4B can combine with and activate CDC7 [31]. As shown in our sequencing data (Table S1), DBF4B was found to be negatively enriched in the sgRNA list (three sgRNAs; *p* < 0.01, log_2_ fold-change < −8). The knockout of DBF4B also downregulates CDC7 phosphorylation activity [32], similar to the effect of XL413 on SCLC. This confirmed that regulating CDC7 activity has potential to improve chemotherapeutic efficacy in SCLC, especially in the case of resistance.

In summary, CDC7 was identified as a synergistic target of chemotherapy in resistant SCLC by using CRISPR/Cas9 screening. The CDC7 inhibitor XL413 aggravated apoptosis and cell cycle arrest in chemo-treated resistant SCLC cells. The combination of XL413 and chemo-therapy showed synergistic effects both in vitro and in vivo. Targeting CDC7 could thus improve the efficacy of chemotherapy in chemo-resistant SCLC.

## Materials and methods

### Cell lines culture and transfection

SCLC cell lines NCI-H69, H69-AR, NCI-H446 were purchased from the American Type Culture Collection (ATCC, USA) and cultured in RPMI-1640 medium (Bionind, Israel), which contained 10% fetal bovine serum (FBS; Procell, China). Cells were cultured in a humidified atmosphere maintaining a 5% CO_2_ level and 37°C. The chemo-resistant subline H446-DDP was established in our laboratory by gradually increasing concentrations of cisplatin (up to 0.5 μg/ml) in the medium of NCI-H446 for 12 months and was maintained with cisplatin. The resistance was evaluated by IC_50_ of cisplatin and etoposide (Fig. S1A). The small interfering RNA (siRNA) for *CDC7* was purchased from RiboBio (China), and transfected into SCLC cell lines using Lipofectamine 3000 (Invitrogen, USA) according to manufacture’s instruction. The *CDC7* siRNA target sequences are as follow: 5′- CAGCTCTGTTTATTTGGCCACAGCA -3′; 5′- ACGCATTCATCAGTTTGGTATTGTT -3′; 5′-GAGAGCCCTGCAGTGAAACTCATGA -3′.

### CRISPR/Cas9 screening and quantification

The human CRISPR knockout Pooled Library (GeCKO v2) Lentivirus (GenScript, China) was used to perform the genome-wide CRISPR knockout screening as described [33]. Shortly, 3 × 10^7^ H69-AR cells per sample were infected by the lentivirus to achieved 250-fold coverage at an MOI of 0.3. Then the cells were selected with 1.5 μg/ml puromycin (Solarbio, China) for 72 h. The day 0 control sample was collected after the puromycin selection. The other cells were treated with either DMSO as negative control or chemo (VP16, 50 μg/ml; DDP, 1 μg/ml) for 7 days. Genomic DNA was extracted using Blood & Cell Culture DNA Kit (Qiagen, Germany), followed by two rounds of PCR as described [34]. The PCR products were purified by QIAquick PCR Purification Kit and QIAquick Gel Extraction Kit (Qiagen, Germany) before next-generation sequencing. Data were analyzed using MAGeCK algorithms [35].

### Chemicals and inhibitor

The Cisplatin (DDP) and Etoposide(VP16)was purchased from Selleck and dissolved in DMSO to a final concentration of 5 mg/ml and 20 mg/ml. The CDC7 inhibitor XL413 was purchased from Selleck and dissolved in ddH_2_O to a final concentration of 10 mM.

### Cell viability, synergy effect and 50% inhibitory concentration (IC_50_)

Cells were seeded in 96-wells plate at appropriate densities and treated with serial of dilutions of drug and incubated for indicated time. CCK-8 regents (Dojindo, Japan) were added as manufacturer’s instruction for 2 h and the absorbance at 450 nm were detected. Final cell viability was calculated relative to the absorbance of negative control cells. The synergy effects between DDP/VP16 and XL413 were determined by SynergyFinder (https://synergyfinder.fimm.fi) based on the highest single agent (HSA) model. The 50% inhibitory concentration (IC_50_) values of the drugs were calculated by GraphPad Prism5 (GraphPad Soft ware Inc., San Diego, CA, USA).

### RNA isolation and quantitative real-time PCR (qRT-PCR)

Total RNA was extracted using TRIzol Reagent (Invitrogen, USA). Reverse transcription was processed using FastKing RT Kit (with gDNase) (Tiangen, China). Quantitative Real-Time PCR (qRT-PCR) was performed using Talent qPCT Premix (SYBR Green) (Tiangen, China) according to the manufacturer’s instructions. The sequences for the forward and reverse primers of β-actin are as follows: forward 5′-CCGTTCCGAAAGTTGCCTTTT-3′ and reverse 5′-ATCATCCATGGTGAGCTGGC-3′. Primers specific for *CDC7*: forward 5′-GGAAAACTGCCAGTTCTTGCCC -3′ and reverse 5′-GGCACTTTGTCAAGACCTCTGG -3′.

The relative quantification was calculated as following: ΔCt [ΔCt = Ct (*CDC7*) − Ct (β-actin)]. Relative expression level was determined as 2^−ΔΔCt^, where ΔΔCt = ΔCt (test samples) −ΔCt (reference samples).

### Antibodies

Antibodies used in this study are as follow: CDC7, MCM2, PARP, Caspase3, cleaved-Caspase3, Bcl-2, Bax, Cyclin E1, Cyclin D1, and GAPDH were purchased from Proteintech Group, UK. Phospho-MCM2 (Ser139) was purchased from Affinity, USA. Chk1 and phospoh-Chk1(Ser 345) was purchased from CST, USA.

### Western blotting assay

Total protein was extracted using RIPA lysis buffer (Beyotime, China) with a protease inhibitor cocktail and phosphatase inhibitor cocktail (Cwbiotech, China). The BCA quantified protein samples were separated by electrophoresis on a SDS-polyacrylamide gel before transfer onto polyvinylidene fluoride (PVDF, Millipore, USA) membranes. After the membranes were blocked with 5% BSA for 1 h in room temperature, they were incubated with specific antibody at 4°C overnight, followed by TBST wash and secondary antibody conjugated to horseradish peroxidase. Next, the signals of the membranes were detected by ECL(enhanced chemiluminescence) Western Blotting Substrate (Pierce, Rockford, IL) according to the manufacturer’s instructions. Full and uncropped western blots was uploaded as supplemental material.

### Cell cycle and apoptosis assay

Cell cycle was detected with Cell cycle detection kit (KeyGen BioTECH, China) as manufacturer’s instructions. Cells were analyzed using flow cytometry. Cell apoptosis was detected with Annexin V-FITC Kit (KeyGen BioTECH, China) using flow cytometric analysis.

### Animal experiments

Twenty Male nude mice aged 4-week were subcutaneously injected with 1*10^7^ H69-AR cells in the flanks. The tumors were measured every two days and the tumor volume was defined as (length*width^2^)/2. When the mean tumor volume reached approximately 100mm^3^, mice were divided into control group, XL413 group, chemo group and XL413 combined with chemo group. Each group contained 5 mice. The treatments contained 3 cycles, 7 days a cycle. DDP was administrated on day1 of each cycle intraperitoneally (2.5 mg/kg), VP16 was administrated on days 1–3 of each cycle intraperitoneally (4 mg/kg), XL413 were administrated on day 1, 3 and 5 of each cycle intraperitoneally (20 mg/kg). The mice were then sacrificed at the end of the third cycle.

### Patient-derived xenograft (PDX) model and Histoculture drug response assay (HDRA)

PDX models were established as described before [15]. HDRA was performed using freshly removed PDX tumor and followed the procedure described before [36]. All regents used in HDRA were purchased from Anticancer, Beijing. Briefly, PDX tumor was divided into 1–2 mm diameter pieces and the viability of tumor specimens were accessed by tetrazoles. Specimens with better viability were picked and paired to reach a close total weight, and each drug combination was performed on 3 pairs of specimens. After 3 days of culture, the viability of the specimens were accessed by MTT. The absorbance per gram of tumor specimens (OD/W) were used to calculate the inhibition rate of each drug combination.

### Statistical analysis

All data analyses were performed using the SPSS statistical software version 22 (Abbott Laboratories, USA) or GraphPad Prism5 (GraphPad Soft ware Inc., USA). Student’s t-test and one-way ANOVA test were performed for comparing differences. *p-*value < 0.05 was considered significant.

## Supplementary information

Supplementary legends

Figure S1. Identifying H69-AR and H446-DDP as chemo-resistant SCLC cell lines.

Figure S2. Silencing CDC7 improves VP16 efficiency in resistant SCLC cells.

Figure S3. XL413 shows synergistic effect with VP16 in resistant SCLC cells.

Figure S4. XL413 shows no synergistic effect with DDP and VP16 in sensitive SCLC cells.

Figure S5. Silencing CDC7 enhances apoptosis induced by chemo-treatment.

Figure S6. Silencing CDC7 and chemo-treatment induce G1/S arrest in resistant SCLC cells.

Table S1 The sgRNA enrichmen between DMSO and chemo-treatment in H69-AR

Figure S7. Full and uncropped western blots

## Data Availability

All data used or analyzed during this study are included in this published article and its supplementary information files.
