# Supplementary material for: Identifying CDC7 as a synergistic target of chemotherapy in resistant small-cell lung cancer via CRISPR/Cas9 screening
Source: Cell Death Discov. 2023 Feb 2;9:40. doi: 10.1038/s41420-023-01315-2 (PMC9892530; doi:10.1038/s41420-023-01315-2)
Supplement: Supplementary file 1 — Supplementary legends [file 41420_2023_1315_MOESM1_ESM.docx]

**Figure S1. Identifying H69-AR and H446-DDP as chemo-resistant SCLC cell lines.**

(A) IC_50_ values of DDP and VP16 in NCI-H69 and H69-AR. (B) IC_50_ values of DDP and VP16 in NCI-H446 and H446-DDP. (C) Expression of *VHL* in SCLC and normal tissues from GSE43346. (D) Expression of *UHRF1* in SCLC and normal tissues from GSE43346.

**Figure S2. Silencing *CDC7* improves VP16 efficiency in resistant SCLC cells.**

(A) IC_50_ of VP16 in H69-AR transfected with scramble or si-*CDC7*. (B) Quantitative of IC_50_ in H69-AR transfected with scramble or si-*CDC7*. (C) IC_50_ of VP16 in H446-DDP transfected with scramble or si-*CDC7*. (D) Quantitative of IC_50_ in H69-AR transfected with scramble or si-*CDC7*. (E, F) Cell viability of H69-AR(E) and H446-DDP(F) transfected with si-*CDC7*-3 using CCK assay.

**Figure S3. XL413 shows synergistic effect with VP16 in resistant SCLC cells.**

(A, B) Quantification of IC_50_ of VP16 in H69-AR(A) and H446-DDP(B) treated with XLl413(50μM, 80μM). (C) Cell viability matrices of VP16 and XL413 in H69-AR. (D) HSA model heatmap of VP16 and XL413 in H69-AR. (E) Cell viability matrices of VP16 and XL413 in H446-DDP. (F) HSA model heatmap of VP16 and XL413 in H446-DDP.

**Figure S4. XL413 shows no synergistic effect with DDP and VP16 in sensitive SCLC cells.**

(A) Quantification of IC_50_ of XL413 in NCI-H69(left) and NCI-H446(right). (B) Quantification of IC_50_ of DDP and VP16 in NCI-H69(left) and NCI-H446(right) treated with XLl413(50μM, 80μM). (C) Cell viability matrices and HSA score of DDP and XL413 in NCI-H69. (D) Cell viability matrices and HSA score of VP16 and XL413 in NCI-H69. (E) Cell viability matrices and HSA score of DDP and XL413 in NCI-H446. (F) Cell viability matrices and HSA score of VP16 and XL413 in NCI-H446.

**Figure S5. Silencing *CDC7* enhances apoptosis induced by chemo-treatment.**

(A) Apoptosis flow cytometry of H69-AR transfected with si-*CDC7* and treated with chemo(1μg/ml DDP, 100μg/ml VP16) single or combined. (B) Apoptosis flow cytometry of H446-DDP transfected with si-*CDC7* and treated with chemo(5μg/ml DDP, 80μg/ml VP16) single or combined. (C) Statistical analysis of apoptotic cell percentage in H69-AR. (D) Statistical analysis of apoptotic cell percentage in H446-DDP.

**Figure S6. Silencing *CDC7* and chemo-treatment induce G1/S arrest in resistant SCLC cells.**

(A) Cell cycle flow cytometry of H69-AR transfected with si-*CDC7* and treated with chemo(1μg/ml DDP, 100μg/ml VP16) single or combined. (B) Quantification of cell percentage in each phase in (A). (C) Cell cycle flow cytometry of H446-DDP transfected with si-*CDC7* and treated with chemo(5μg/ml DDP, 80μg/ml VP16) single or combined. (D) Quantification of cell percentage in each phase in (C).

**List of Supporting Information**:

Supplementary Table 1 The sgRNA enrichment between DMSO and chemo-treatment in H69-AR

Figure S1 Identifying H69-AR and H446-DDP as chemo-resistant SCLC cell lines.

Figure S2 Silencing *CDC7* improves VP16 efficiency in resistant SCLC cells.

Figure S3 XL413 shows synergistic effect with VP16 in resistant SCLC cells.

Figure S4 XL413 shows no synergistic effect with DDP and VP16 in sensitive SCLC cells.

Figure S5 Silencing *CDC7* enhances apoptosis induced by chemo-treatment.

Figure S6 Silencing *CDC7* and chemo-treatment induce G1/S arrest in resistant SCLC cells.

Figure S7. Full and uncropped western blots.
