# Supplementary figures and images for: Identifying CDC7 as a synergistic target of chemotherapy in resistant small-cell lung cancer via CRISPR/Cas9 screening
Source: Cell Death Discov. 2023 Feb 2;9:40. doi: 10.1038/s41420-023-01315-2 (PMC9892530; doi:10.1038/s41420-023-01315-2)

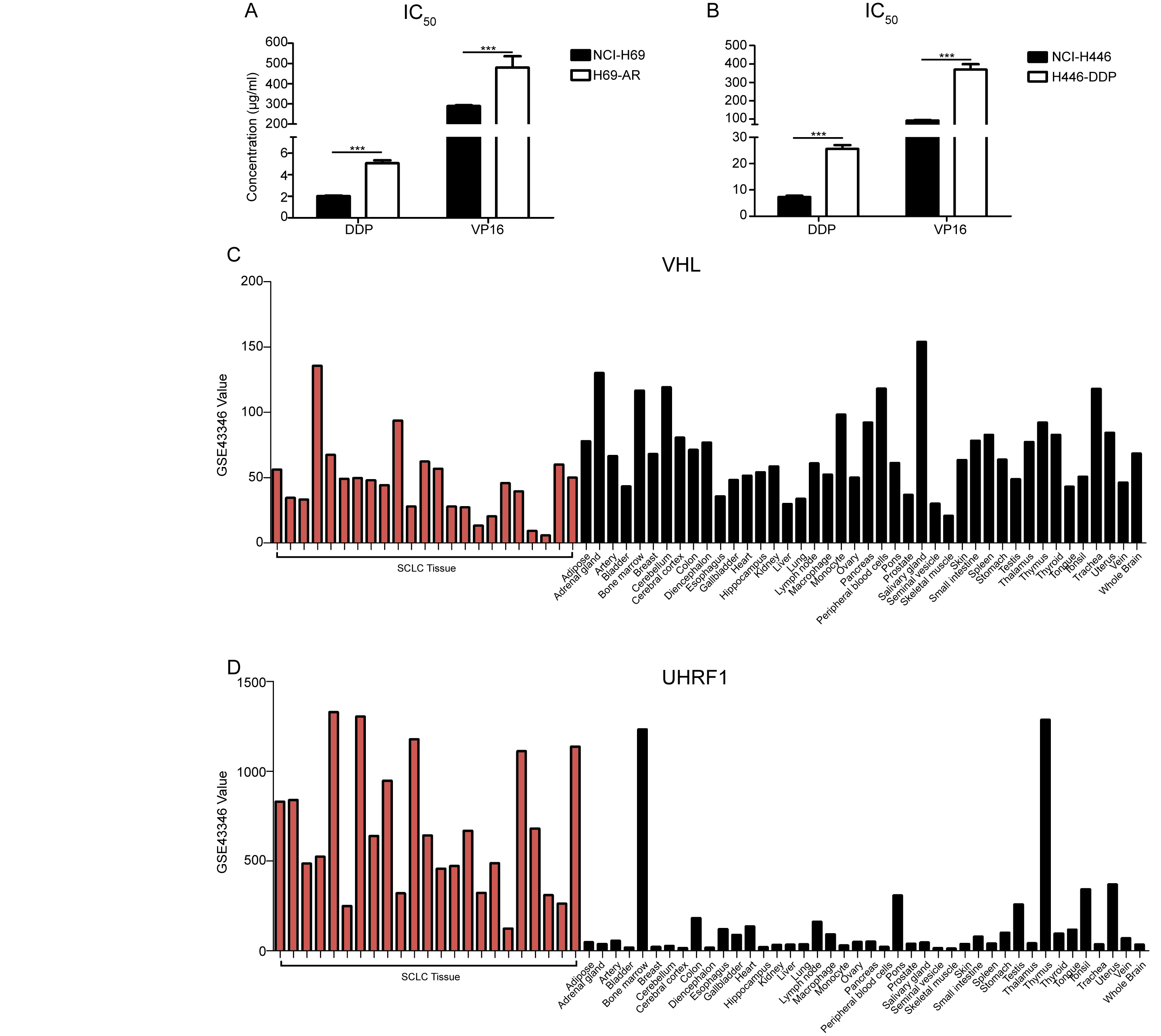

Supplement: Supplementary file 2 — Figure S1. Identifying H69-AR and H446-DDP as chemo-resistant SCLC cell lines. [file 41420_2023_1315_MOESM2_ESM.tif]

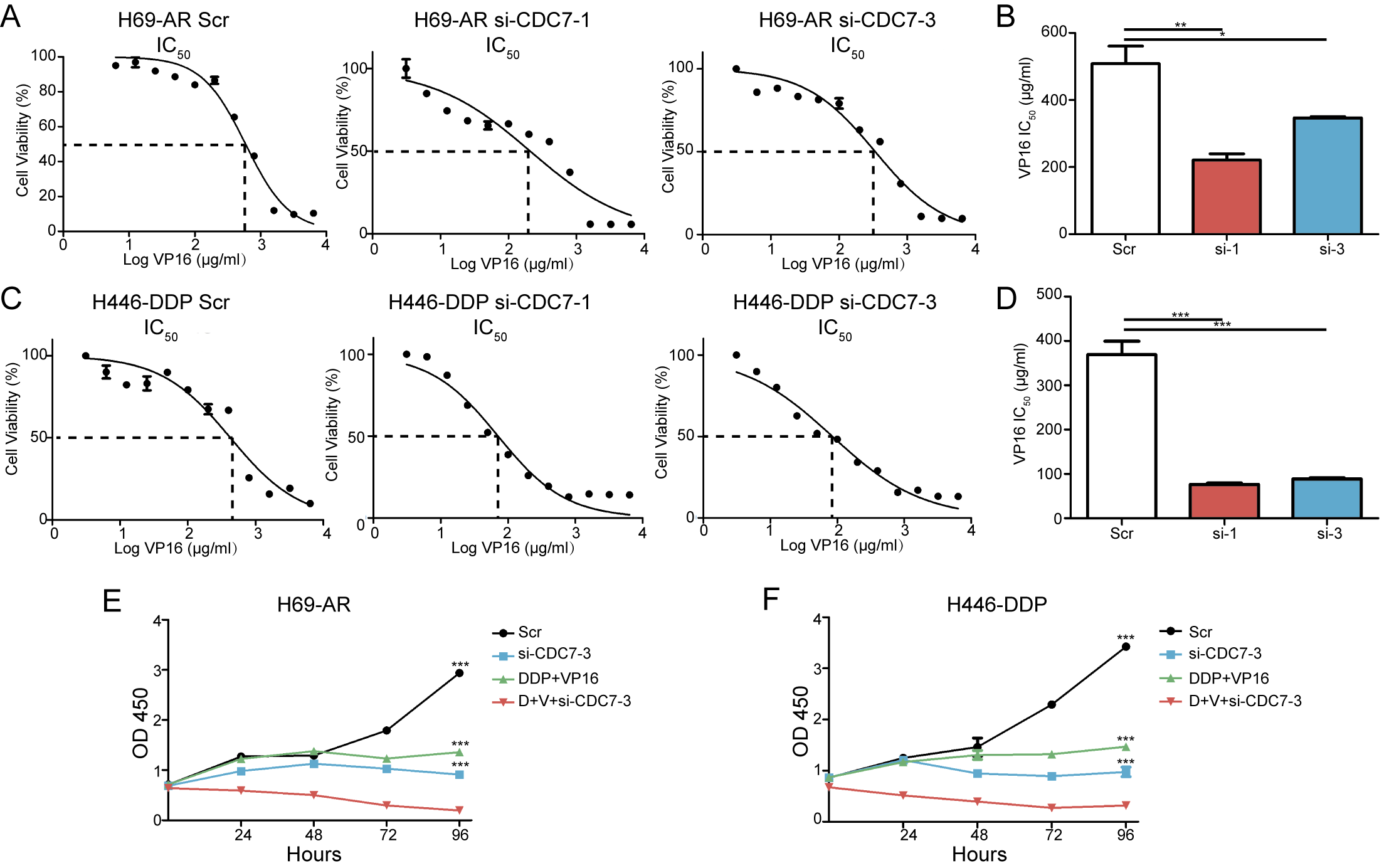

Supplement: Supplementary file 3 — Figure S2. Silencing CDC7 improves VP16 efficiency in resistant SCLC cells. [file 41420_2023_1315_MOESM3_ESM.tif]

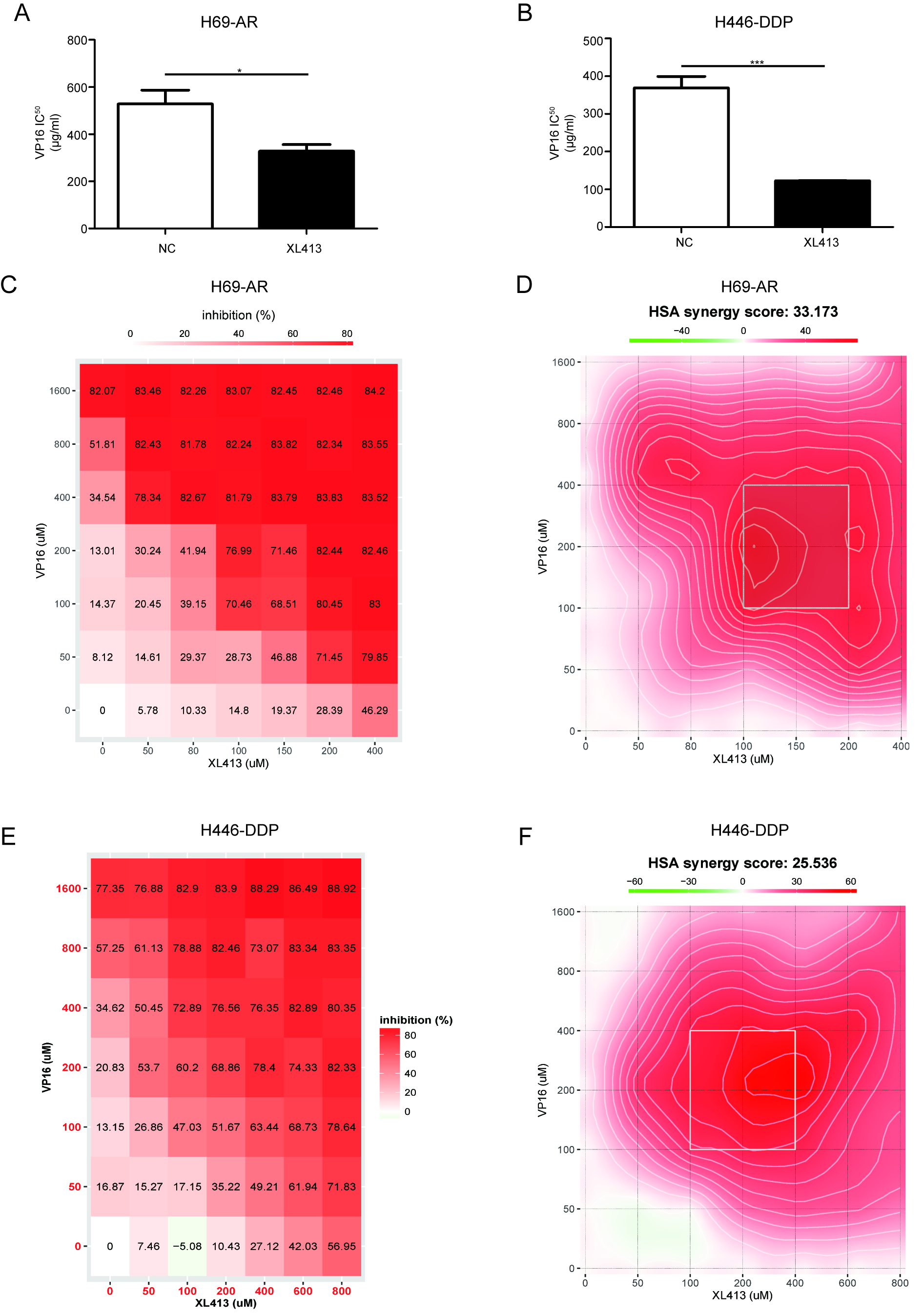

Supplement: Supplementary file 4 — Figure S3. XL413 shows synergistic effect with VP16 in resistant SCLC cells. [file 41420_2023_1315_MOESM4_ESM.tif]

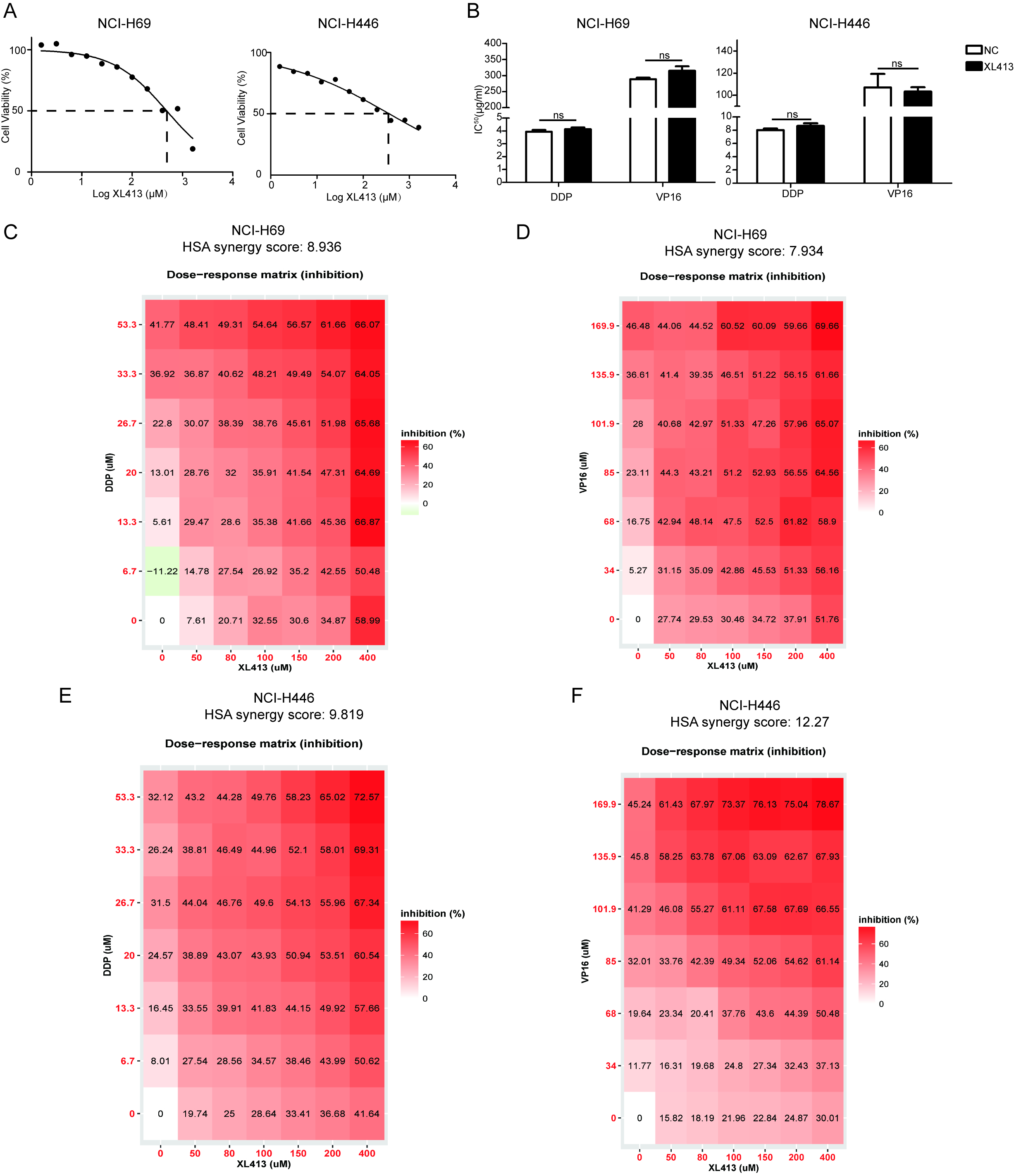

Supplement: Supplementary file 5 — Figure S4. XL413 shows no synergistic effect with DDP and VP16 in sensitive SCLC cells. [file 41420_2023_1315_MOESM5_ESM.tif]

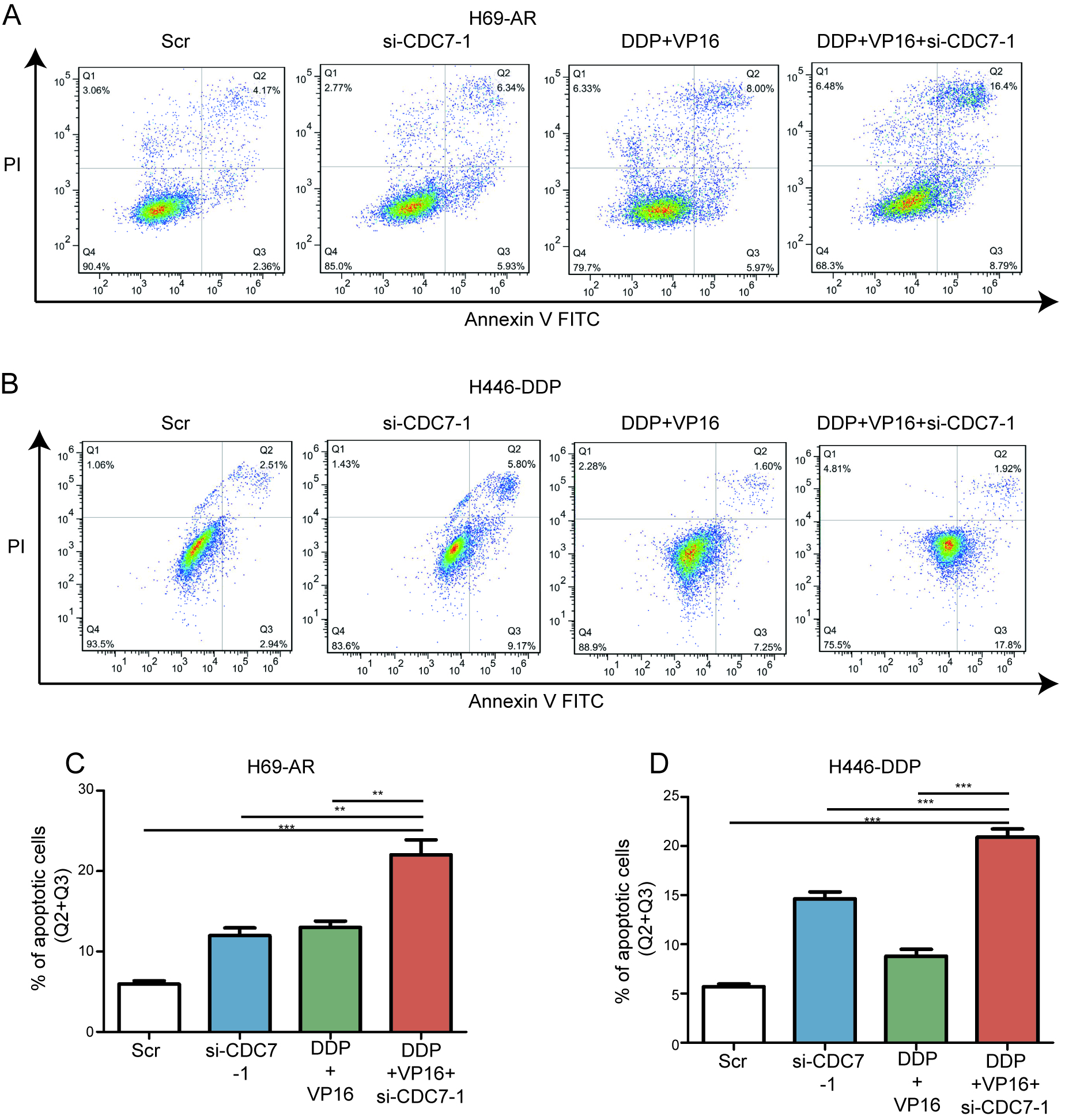

Supplement: Supplementary file 6 — Figure S5. Silencing CDC7 enhances apoptosis induced by chemo-treatment. [file 41420_2023_1315_MOESM6_ESM.tif]

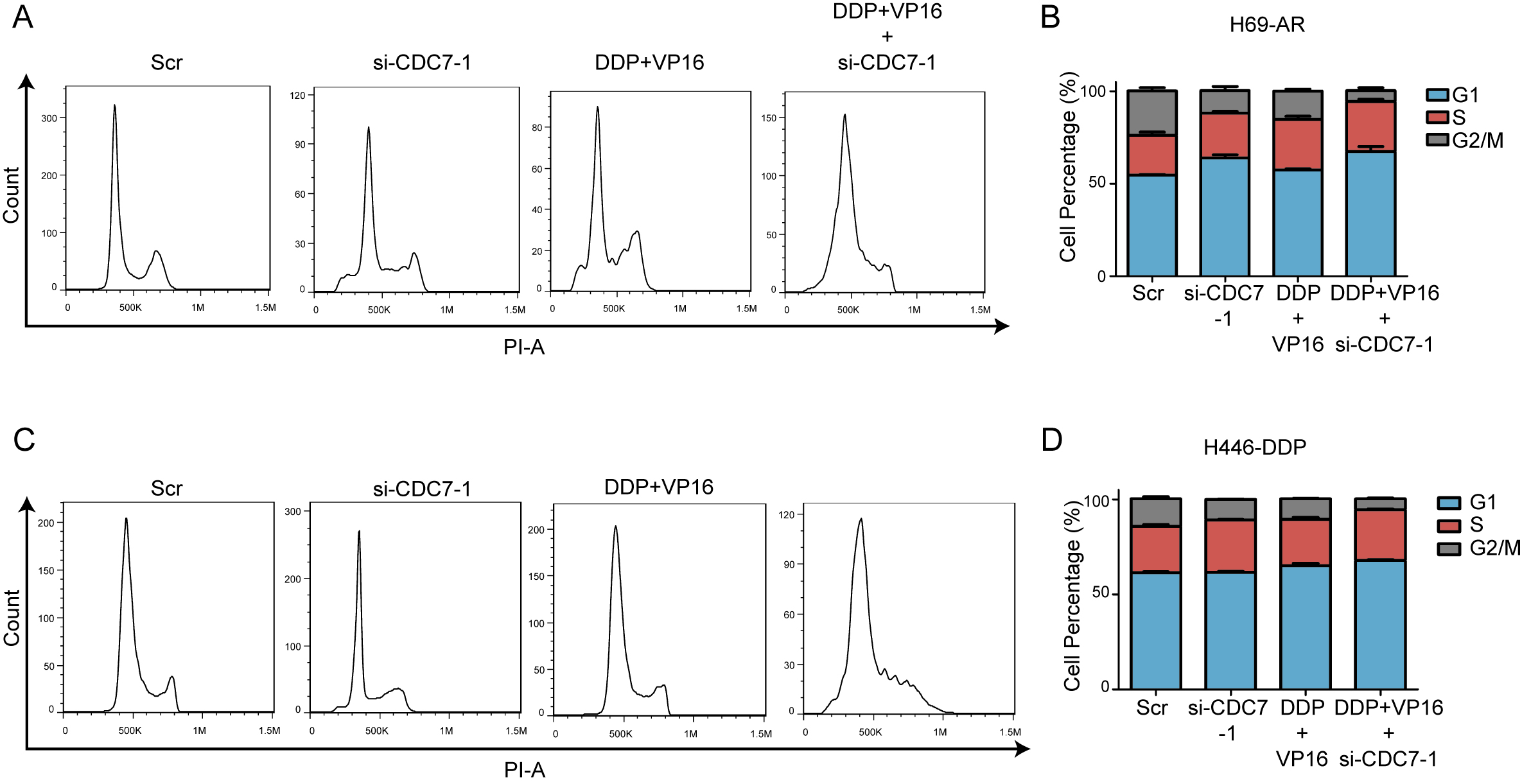

Supplement: Supplementary file 7 — Figure S6. Silencing CDC7 and chemo-treatment induce G1/S arrest in resistant SCLC cells. [file 41420_2023_1315_MOESM7_ESM.tif]
