# Supplementary material for: Identifying CDC7 as a synergistic target of chemotherapy in resistant small-cell lung cancer via CRISPR/Cas9 screening
Source: Cell Death Discov. 2023 Feb 2;9:40. doi: 10.1038/s41420-023-01315-2 (PMC9892530; doi:10.1038/s41420-023-01315-2)

Figure 2 Western blot original images

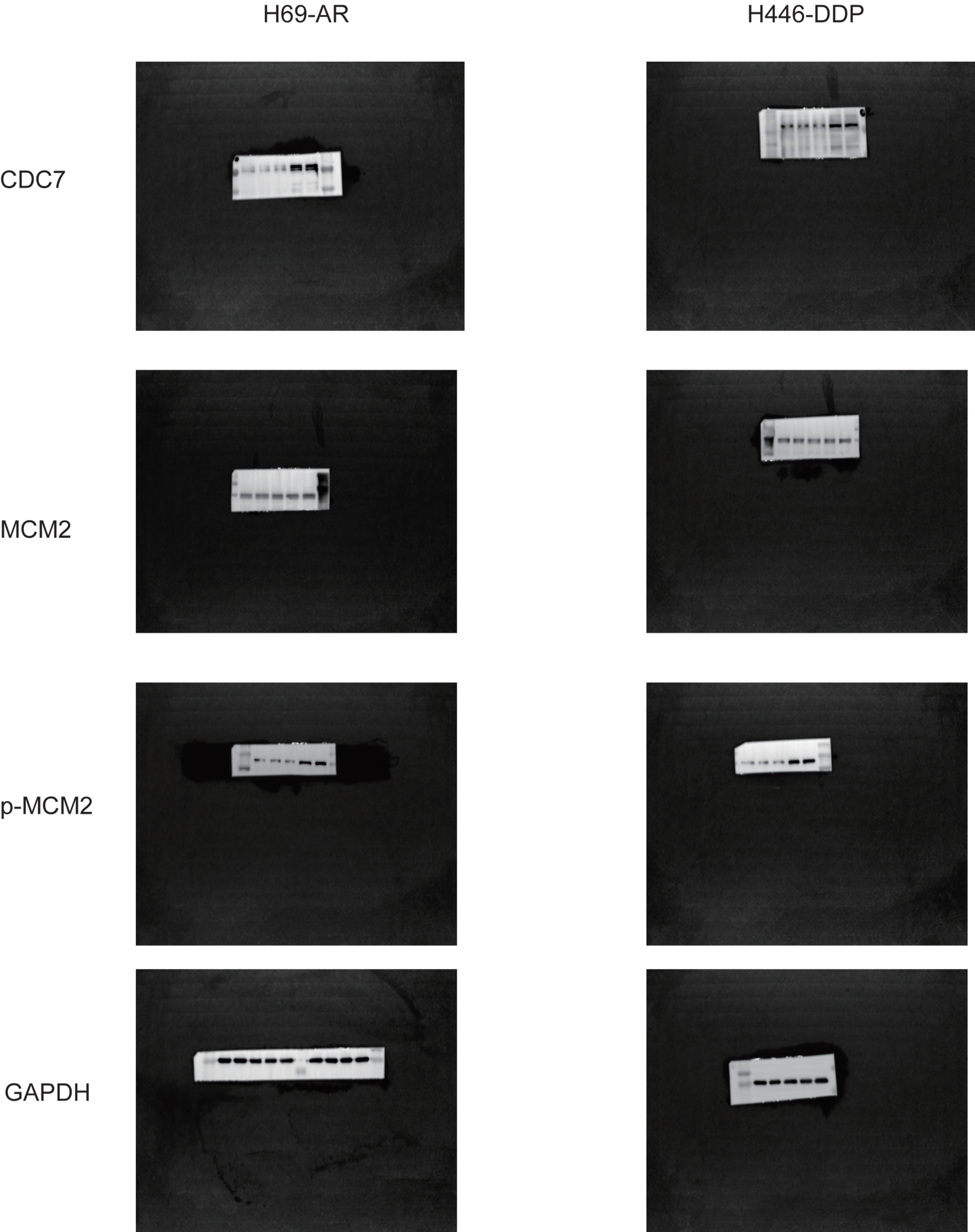

Figure 4 Western blot original images

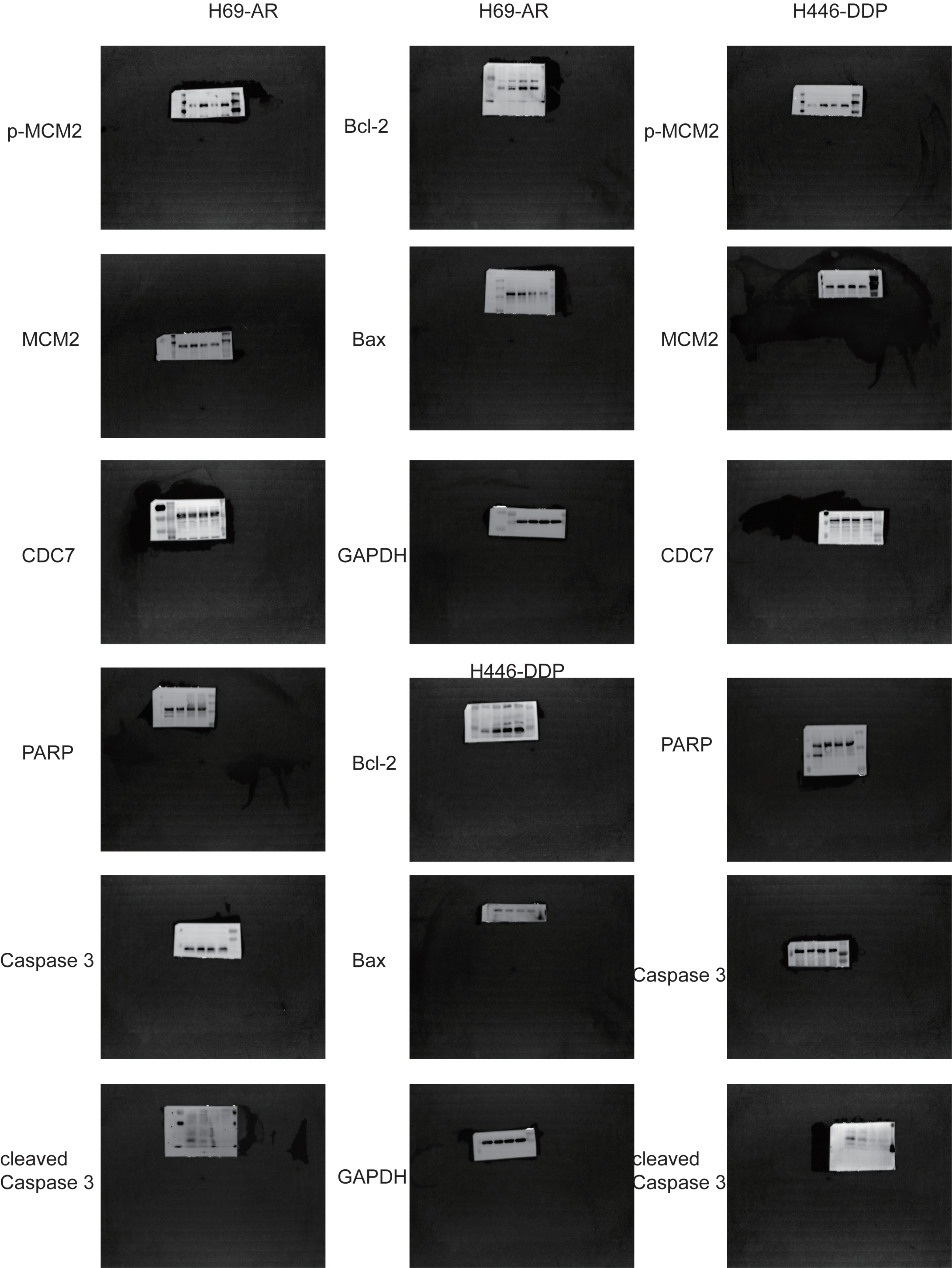

Figure 5 Western blot original images

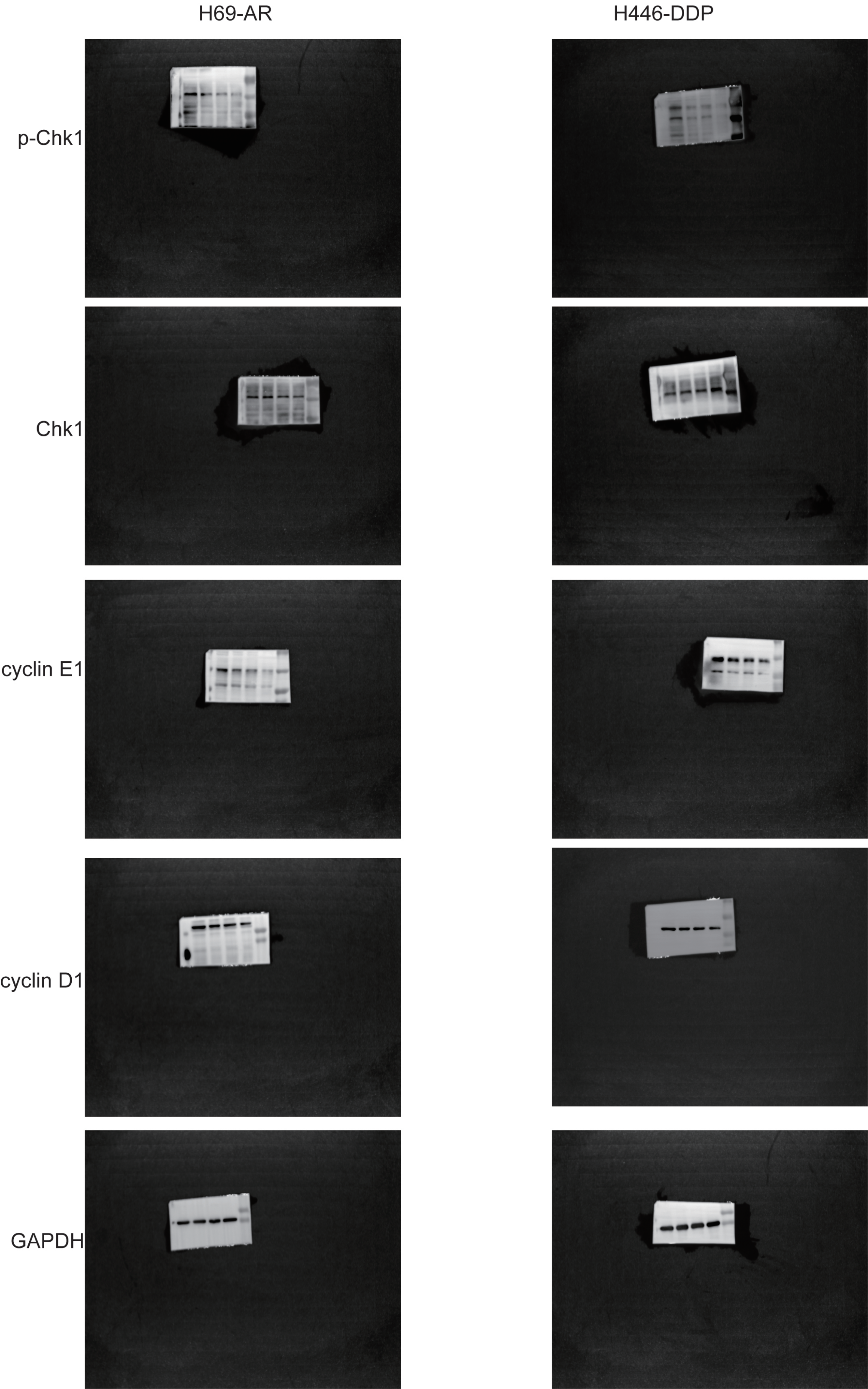

Supplement: Supplementary file 9 — Figure S7. Full and uncropped western blots [file 41420_2023_1315_MOESM9_ESM.pdf]
